# Prognostic value of perfusion cardiovascular magnetic resonance with adenosine triphosphate stress in stable coronary artery disease

**DOI:** 10.1186/s12968-021-00770-z

**Published:** 2021-06-24

**Authors:** Ming-Yen Ng, Chi Yeung Chin, Pui Min Yap, Eric Yuk Fai Wan, JoJo Siu Han Hai, Stephen Cheung, Hung Fat Tse, Chiara Bucciarelli-Ducci, Dudley John Pennell, Kai-Hang Yiu

**Affiliations:** 1 Department of Diagnostic Radiology, The University of Hong Kong, Hong Kong, China; 2 Department of Medical Imaging, The University of Hong Kong-Shenzhen Hospital, Shenzhen, China; 3 Department of Family Medicine and Primary Care, The University of Hong Kong, Hong Kong, China; 4 Cardiology Division, Department of Medicine, The University of Hong Kong, Hong Kong, China; 5 Department of Radiology, Queen Mary Hospital, Hong Kong, China; 6 Royal Brompton and Harefield NHS Foundation Trust, London, UK; 7 Imperial College, London, UK; 8 Department of Cardiology, The University of Hong Kong-Shenzhen Hospital, Shenzhen, China; 9 Department of Diagnostic Radiology, The University of Hong Kong, Queen Mary Hospital, Room 406, 4/F Block K102 Pokfulam Road, Hong Kong, Hong Kong SAR, China

**Keywords:** Adenosine triphosphate, Stress, Cardiovascular magnetic resonance, Prognosis, Coronary artery disease

## Abstract

**Background:**

Adenosine triphosphate (ATP) has been predominantly used in the Asia–Pacific region for stress perfusion cardiovascular magnetic resonance (CMR). We evaluated the prognosis of patients stressed using ATP, for which there are no current data.

**Methods:**

We performed a retrospective longitudinal study from January 2016 to December 2020 and included 208 subjects with suspected obstructive coronary artery disease (CAD) who underwent ATP stress perfusion CMR. An inducible stress perfusion defect was defined as a subendocardial dark rim involving ≥ 1.5 segments that persisted for ≥ 6 beats during stress but not at rest. The primary outcome measure was a composite of major adverse cardiovascular events (MACE) including (1) cardiac death, (2) nonfatal myocardial infarction, (3) cardiac hospitalization, (4) late coronary revascularization. We compared outcomes in patients with and without perfusion defect using Kaplan–Meier and log rank tests. Significant predictors of MACE were identified using multivariable Cox regression analysis.

**Results:**

Median follow-up was 3.3 years. Patients with no stress perfusion defect had a lower incidence of MACE (p < 0.001), including lower cardiac hospitalization (p = 0.004), late coronary revascularization (p = 0.001) and cardiac death (p = 0.003). Significant independent predictors for MACE were stress induced perfusion defect (p < 0.001, hazard ratio [HR] = 3.63), lower left ventricular ejection fractino (LVEF) (p < 0.001, HR = 0.96) and infarct detected by late gadolinium enhancement (LGE) (p = 0.001, HR = 2.92).

**Conclusion:**

Perfusion defects on ATP stress are predictive of MACE which is driven primarily by cardiac hospitalization, late coronary revascularization and cardiac death. Significant independent predictors of MACE were stress induced perfusion defect, lower LVEF and infarct detected by LGE.

**Supplementary Information:**

The online version contains supplementary material available at 10.1186/s12968-021-00770-z.

## Introduction

Stress perfusion cardiac magnetic resonance (CMR) is a low-risk and non-invasive imaging modality for diagnosis of coronary artery disease (CAD) with high sensitivity, specificity and accuracy [1, 2]. Apart from its diagnostic accuracy, stress perfusion CMR is also recognized for its high prognostic value in risk stratification of patients of known or suspected CAD when using adenosine, dipyridamole and regadenoson as the vasodilator agent [3–10]. However, the prognostic value of adenosine triphosphate (ATP) as a vasodilator for stress CMR is not well-established. ATP has similar vasodilatory and hemodynamic changes to adenosine [11] and due to its lower cost and/ or licensing/ production issues of alternative pharmaceutical agents, it has been a commonly used alternative in the Asian Pacific region [11–14] and some European countries [15, 16]. Although ATP stress CMR might be assumed to have prognostic significance, this has never been demonstrated. Therefore, we performed this study to evaluate the prognostic significance of ATP stress CMR in order to confirm this hypothesis.

## Methods

**Fig. 1:**
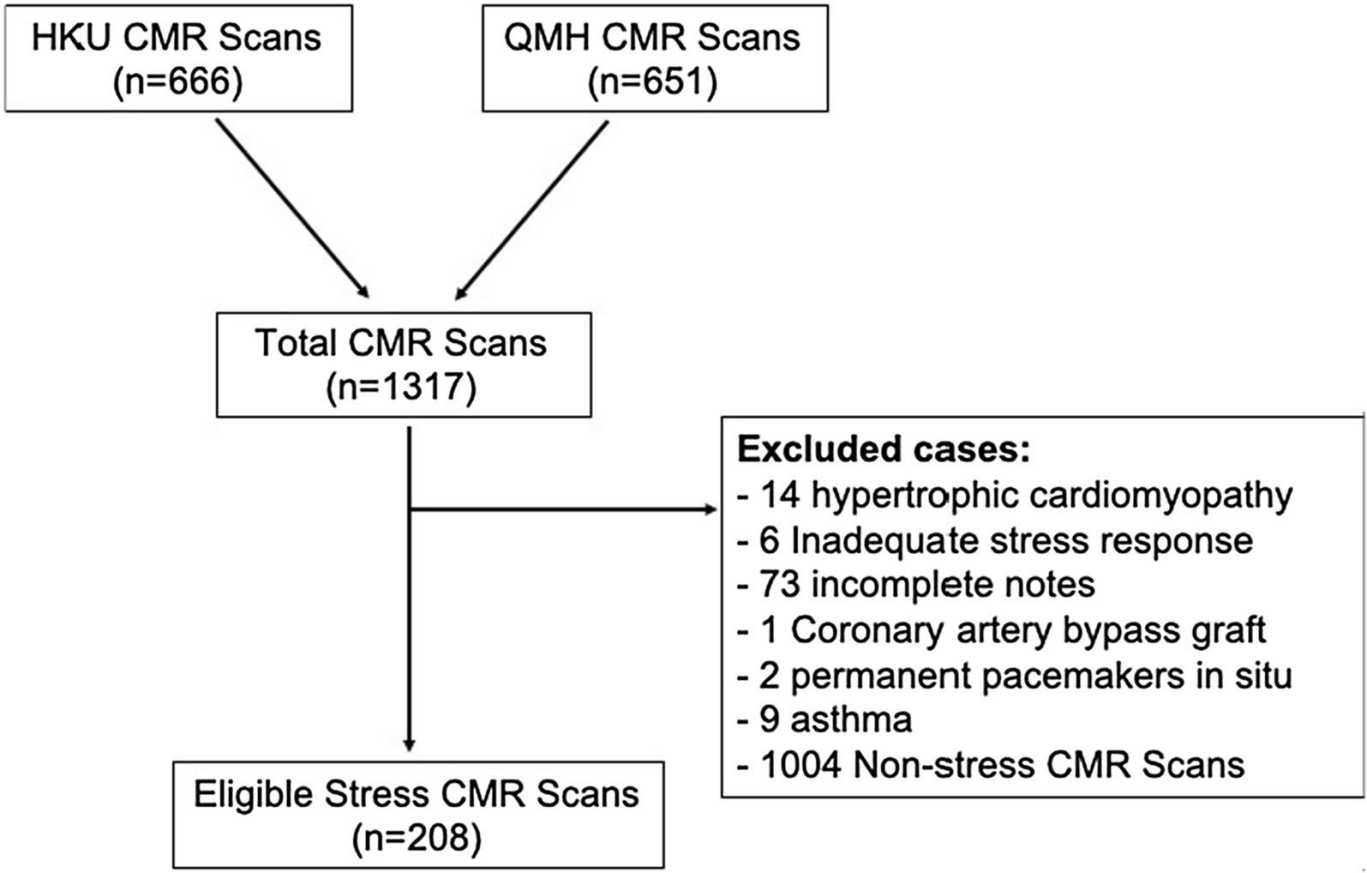
Patient flow diagram

This study was approved by the Institutional Review Board of the Hong Kong West Cluster. Requirement for informed consent was waived. This study was a retrospective longitudinal study. Patients from the University of Hong Kong and Queen Mary Hospital’s database were identified from 1^st^ January 2016 to 31st March 2019 and 1st January 2017 to 31^st^ December 2017 respectively. Inclusion criteria were patients ≥ 18 years undergoing ATP stress CMR for suspected or known obstructive CAD. Exclusion criteria included coronary artery bypass grafts, known hypertrophic cardiomyopathy, myocarditis, implantation of cardiac pacemaker or implantable cardiac defibrillator, history of asthma or bronchospasm, incomplete notes to determine adequate stress and second or third-degree atrioventricular block. A total of 208 subjects were identified (see Fig. 1).

### CMR protocol

A 3 T CMR scanner (Achieva, Philips Healthcare, Best, the Netherlands) with a 16-element phased array coil or a 1.5 T CMR scanner (Aera, Siemens Healthineers, Erlangen, Germany) with a 32-element phased array coil were used in all cases. Subjects were given ATP at an infusion rate of 0.14 mg/kg/min for at least 3 min, followed by an intravenous administration of gadoterate meglumine (injection rate: 3 to 4 mL/s, with a subsequent 30 mL saline flush at the same flow rate) to obtain the first-pass perfusion images using a T1 weighted fast gradient echo sequence for both scanners. [Philips Achieva: echo time (TE) 1.2 ms, repetition time (TR) 2.5 ms, flip angle 20°, field of view 320 mm x 320 mm, slice thickness 10 mm, Siemens Aera: TE 0.98 ms, TR 177 ms, flip angle 50°, voxel size 2.3 × 2.3 x 8 mm]. Three perfusion short-axis slice images (base, mid, apex) of the left ventricle were acquired. This was followed by acquisition of a short-axis cine stack using balanced steady-state free precession (bSSFP) (3 T Philips Achieva: TE/TR = 1.48/2.96 ms, flip angle 45°, slice thickness 8 mm, 25 cardiac phases; 1.5 T Siemens Aera TE/TR 1.28/40.17 ms, voxel size 1.2 × 1.2x6mm. flip angle 62°, slice thickness 8 mm, 25 phases) and analyzed with cmr42 software (Circle Cardiovascular Imaging, Inc., Calgary, Alberta, Canada) or Syngo Via (Siemens Healthineers). Long axis bSSFP cine images were acquired in the 2, 3 and 4-chamber orientations. Rest perfusion images were acquired in the same three short axis positions as the stress perfusion images at least 10 min after termination of ATP infusion. Inversion time scout images were acquired to determine the ideal inversion time for late gadolinium enhancement (LGE). LGE images were acquired 8–15 min after the second gadoterate meglumine injection for rest perfusion images. For the 3 T Philips Achieva, segmented phase sensitive inversion recovery (PSIR) LGE images were acquired (TE 3 ms, TR 6.1 ms, flip angle 25 degrees, slice thickness 8 mm). For the 1.5 T Siemens Aera, PSIR LGE images were acquired (TE 3–4 ms, TR 8–9 ms, flip angle 25 degrees, slice thickness 8 mm).

### Adequate stress response

Adequate stress response to ATP was defined as two or more of the following criteria: (1) heart rate increase ≥ 10 bpm, (2) systolic blood pressure decrease ≥ 10 mmHg, (3) positive splenic switch-off sign, and (4) presence of stress symptoms (e.g. chest pain, shortness of breath, headache). Inadequate stress was defined as 0 or 1 of the above criteria. A subsequent 50% increased infusion rate would be given for an inadequate stress response [17]. If adequate stress response was still not achieved, no further infusion rate increase was delivered.

### ATP perfusion and LGE assessment

**Fig. 2:**
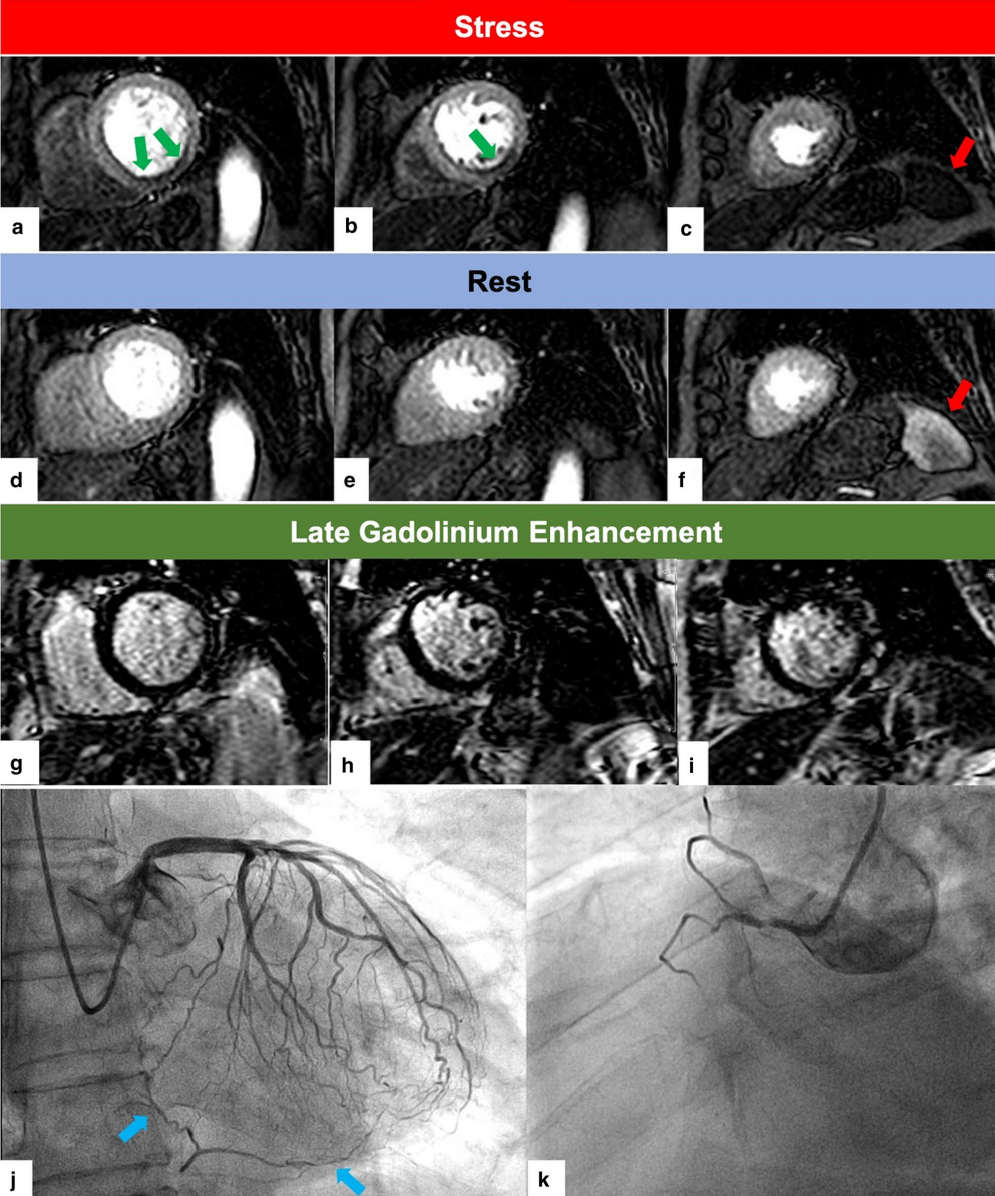
Case of patient undergoing adenosine triphosphate (ATP) stress cardiovascular magnetic resonance (CMR). Stress perfusion (**a**–**c**), rest perfusion (**d**–**f**), late gadolinium enhancement (LGE) (**g**–**i**), left coronary artery catheter angiogram (G) and right coronary artery (RCA) catheter angiogram (H) images are illustrated. Stress induced perfusion defects (green arrows) are demonstrated in the left ventricular (LV) inferior wall on the basal and mid-ventricular slices (**a**, **b**) which resolves at rest (**d**, **e**). The left coronary artery catheter angiogram (**j**) shows collateral vessels coming from the left anterior descending coronary artery (LAD) and left circumflex coronary artery (LCX) to perfuse the RCA branches. LGE images (**g**–**l**) show no evidence of infarction. RCA coronary angiogram (**k**) shows the RCA is occluded. Note, that the splenic switch-off sign is present (red arrows in **c,** **f**) with the spleen unenhanced during stress and the spleen enhancing during rest

Following previous publications on prognostic significance of regadenoson, adenosine and dipyridamole stress CMR [7, 8, 18], we identified significant inducible stress perfusion defects on the stress perfusion images (see Fig. 2) as previously described [19]. Briefly a stress-induced perfusion defect was defined as a subendocardial rim of reduced signal involving ≥ 1.5 segments that persisted for ≥ 6 beats during stress but not at rest without matching enhancement on LGE imaging [19, 20]. Rest perfusion defects and perfusion defects matching LGE were not regarded as stress induced perfusion defects. Reporting radiologists and cardiologists were blinded to the clinical outcome. Reporting was performed by either one or two radiologists/ cardiologists. At a minimum one of those reporting had level 3 accreditation. Myocardial LGE was quantified using the cmr42 software (Circle Cardiovascular Imaging, Inc.) [21]. LGE was identified as 5 standard deviations above the mean.

### CMR ventricular function, volume analysis and image quality assessment

Left ventricular (LV) function and volumes were assessed using the bSSFP short axis cine images and analyzed with cmr42 software (Circle Cardiovascular Imaging, Inc.) to give the following CMR parameters: (1) LV end-diastolic volume, (2) LV end-systolic volume, (3) LV ejection fraction (LVEF), and (4) LV mass. Volumes and mass were corrected for body surface area using the Mosteller equation [22]. Image quality scoring of the perfusion and LGE images were performed using a Likert scale from 1 to 4. A score of 1 being excellent and 4 being non-diagnostic. Fifty cases were chosen at random. Mean and standard deviation for perfusion and LGE image quality scoring were 1.4 (SD 0.6) and 1.8 (SD 0.6).

### Major adverse cardiovascular events

The subsequent hospital-related activities of the subjects were obtained through the territory-wide Electronic Patient Record system, including any clinical follow-ups, inpatient and outpatient care records, and examinations performed. The primary outcome measure of this study is a composite of major adverse cardiovascular evvents (MACE) consisting of (1) cardiac death, (2) non-fatal myocardial infarction (MI), (3) cardiac hospitalization, and (4) late coronary revascularization. Cardiac hospitalization included any in-patient hospital stay due to a cardiovascular events (i.e. heart failure or acute coronary syndrome), while late coronary revascularization includes percutaneous coronary intervention, and coronary artery bypass grafting more than 90 days post stress CMR. We recorded all cardiovascular events these subjects experienced, and their first events were used for analysis regarding composite MACE. For the annualized event rate, we used cardiac death and non-fatal myocardial infarct events only in keeping with other publications and meta-analysis [8, 10].

### Statistical analysis

Continuous variables are presented as mean ± standard deviations. Categorical variables are presented in numbers with percentages in brackets. Student’s t-test was used to compare normally distributed variables. Mann–Whitney U test was used to compare non-normally distributed variables. Categorical variables were compared using Fisher’s exact test.

The outcomes of subjects with and without inducible perfusion defects on ATP stress CMR findings were compared using Kaplan Meier survival curve with log rank test. Sub-analysis of each type of MACE was also performed with Kaplan Meier survival curve and log rank test to determine the main drivers of the composite MACE outcome. A multivariable Cox regression model was created using the variables stress induced perfusion defect, LGE infarct and LVEF which all had a p-value < 0.05 on univariate Cox regression analysis. All statistical analyses were done using SPSS (version 26.0, Statistical Package for the Social Sciences, International Business Machines, Inc., Armonk, New York, USA).

## Results

Subjects had a mean age of 61.2 ± 14.8 years and 123 were male (59.1%). Table 1 shows the characteristics of subjects with MACE compared to those without.

**Table 1:** Patient characteristics of study population (MACE vs without MACE)

	Subjects with MACE (n = 39)	Subjects without MACE (n = 169)	P value
*General information*			
Age (yrs)	67.8 ± 12.8	59.8 ± 14.8	0.001*
Male	26 (66.7%)	97 (57.4%)	0.288
Height (cm)	163.8 ± 10.4	163.5 ± 9.7	0.847
Weight (kg)	67.5 ± 13.3	67.3 ± 14.6	0.932
BMI (m^2^)	25.0 ± 3.8	25.0 ± 4.2	0.988
Hypertension	26 (66.7%)	90 (53.3%)	0.128
Diabetes	11 (28.2%)	42 (24.9%)	0.665
Hyperlipidemia	14 (35.9%)	64 (37.9%)	0.819
Smoking	9 (23.1%)	11 (6.5%)	0.002*
Estimated glomerular filtration rate (mL/min/1.73m^2^)	73.2 ± 23.8	80.5 ± 21.3	0.025*
Atrial fibrillation	8 (20.5%)	6 (3.6%)	< 0.001*
*Cardiac history*			
Heart failure	5 (12.8%)	6 (3.6%)	0.020*
Myocardial infarction	2 (5.1%)	0 (0%)	0.003*
Coronary artery disease	13 (33.3%)	58 (34.3%)	0.907
*Symptoms for CMR referral*			
Chest pain	16 (41.0%)	68 (40.2%)	0.928
Shortness of breath	0	8 (4.7%)	0.166
Palpitation	0	6 (3.6%)	0.232
Dizziness	0	2 (1.2%)	0.495
Loss of consciousness	1 (2.6%)	3 (1.8%)	0.746
*CMR parameters*			
1.5 T	16 (41.0%)	71 (42.0%)	0.910
3.0 T	23 (59.0%)	98 (58.0%)	0.910
LV end-diastolic volume index(mL/m^2^)	101.1 ± 64.1	81.3 ± 23.1	0.001*
LV end-systolic volume index(mL/m^2^)	60.4 ± 65.4	33.8 ± 21.0	< 0.001*
LV ejection fraction (%)	50.2 ± 19.5	60.7 ± 12.6	< 0.001*
LV mass index (g/m^2^)	83.7 ± 39.7	60.9 ± 17.0	< 0.001*
Heart rate at rest (bpm)	76.4 ± 16.6	67.2 ± 12.3	< 0.001*
Systolic blood pressure at rest (mmHg)	146.2 ± 22.7	139.9 ± 20.6	0.103
Diastolic blood pressure at rest (mmHg)	89.3 ± 12.1	85.1 ± 13.1	0.241
ATP infusion time (min)	4.3 ± 0.7	4.4 ± 0.8	0.496
Abnormal wall motion	17 (43.6%)	22 (13.0%)	< 0.001*
*Medications*			
Beta-blocker	20 (51.3%)	68 (40.2%)	0.208
Calcium channel blocker	9 (23.1%)	52 (30.8%)	0.342
ACE inhibitor	15 (38.5%)	37 (21.9%)	0.031*
Statin	27 (69.2%)	96 (56.8%)	0.155
Aspirin	21 (53.9%)	81 (47.9%)	0.505
Digoxin	0 (0%)	1 (0.6%)	0.630
*Side-effects*			
Chest pain	13 (33.3%)	51 (30.2%)	0.700
Shortness of breath	10 (25.6%)	31 (18.3%)	0.302
Headache	3 (7.7%)	14 (8.3%)	0.903
Palpitation	3 (7.7%)	12 (7.1%)	0.898
Hot flushing	0 (0%)	2 (1.2%)	0.495
*Stress CMR findings*			
LVEF < 50%	15 (38.5%)	20 (11.8%)	< 0.001*
Myocardial LGE	21 (53.9%)	30 (17.8%)	< 0.001*
LGE (%)	2.95 ± 7.13	1.15 ± 4.63	0.056
Stress Induced Perfusion Defect	17 (43.6%)	21 (12.4%)	< 0.001*

Data is presented as mean ± standard deviation or count with percentage in brackets

*ATP* adenosine triphosphate, *ACE* angiotensin converting enzyme, *BMI* body mass index, *LV* Left ventricle, *LGE* Late gadolinium enhancement; * = p < 0.05

Adequate stress response to standard dose and a 50% higher infusion rate was achieved in 196 (94.2%) and 12 (5.8%) cases respectively.

### ATP side-effects

One hundred patients (48.1%) experienced symptoms during ATP infusion. Side-effects included chest pain, shortness of breath, headache, palpitation and hot flushing. The symptoms were mild and resolved shortly after the ATP infusion was completed. No medical complications were encountered. See Table 2 for the frequency of different side-effects experienced by subjects during stress test.

**Table 2:** Frequency of ATP side-effects experienced by subjects during stress test

	Chest pain	Shortness of breath	Headache	Palpitation	Hot flushing
Frequency	64	41	17	15	2

### Abnormal CMR findings

Out of 208 patients, 87 patients (male: female = 59:28) had abnormal CMR findings. Of these 87 patients, the patients had one or more of the following abnormalities: 35 (40.2%) had LVEF < 50%, 51 (58.6%) had MI detected by LGE, and 38 (43.7%) had stress induced perfusion defects. 6 patients (6.9%) had all three abnormalities, 25 (28.7%) had two of the three abnormalities, and 56 (64.4%) had only one of the three abnormalities.

### Incidence of MACE composite endpoints

The median follow-up period was 3.3 years (interquartile range from 2.7 to 3.7 years).

**Fig. 3:**
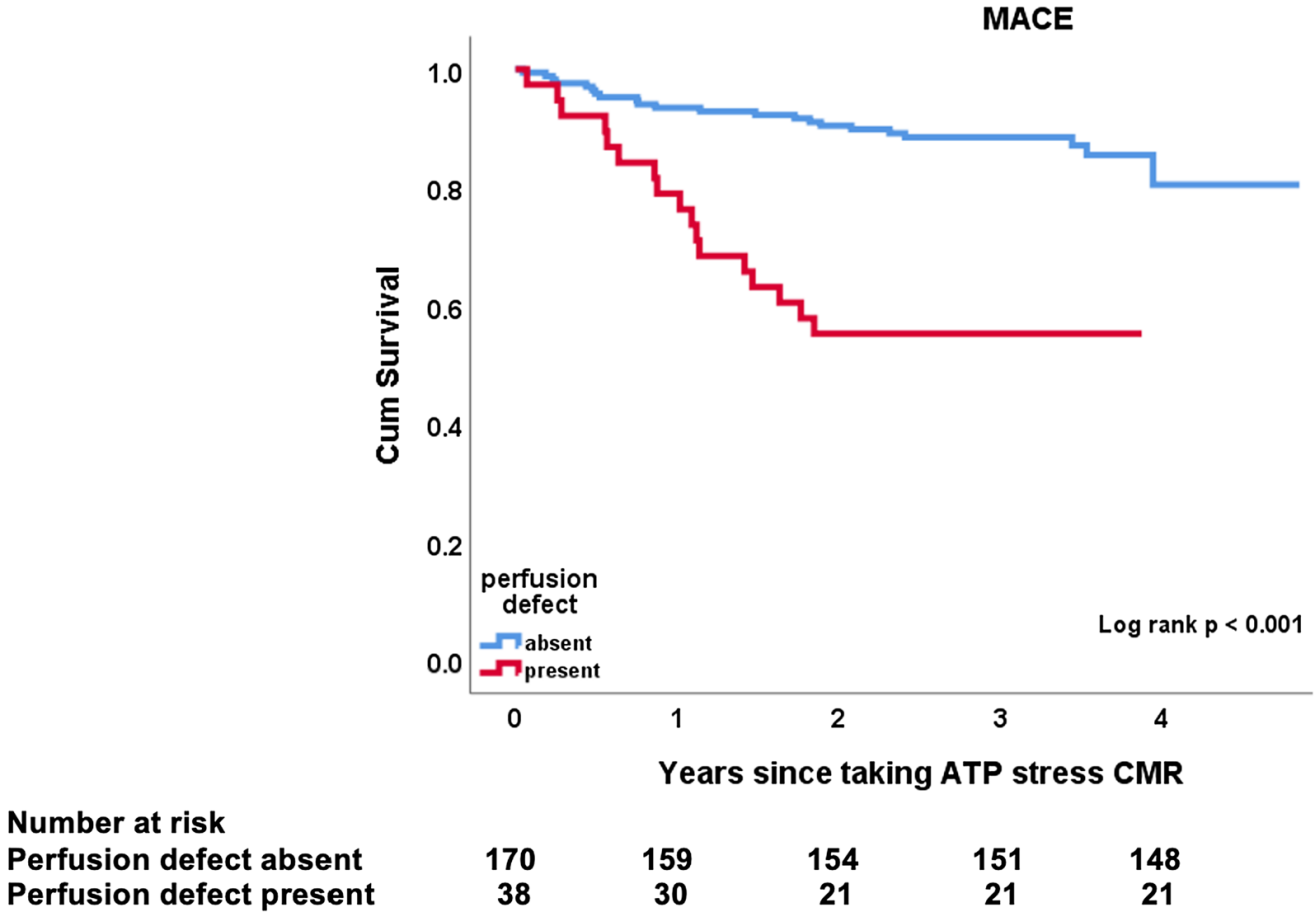
Incidence of composite major adverse cardiovascular events (MACE) between normal and abnormal stress test findings. Estimated cumulative incidence of composite MACE was significantly higher in subjects with abnormal stress findings (log rank p-value < 0.001)

**Fig. 4:**
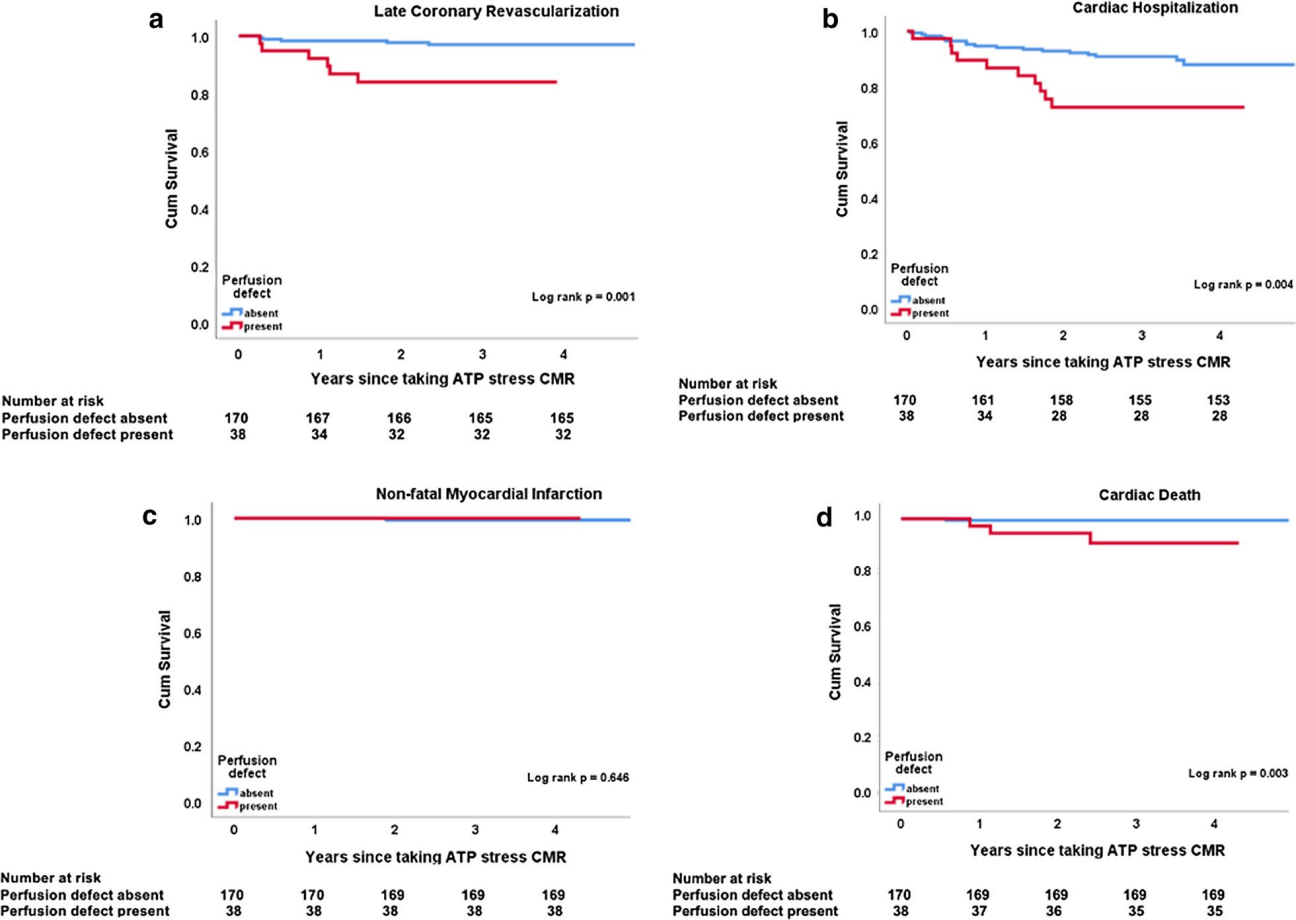
Sub-analysis of the four parameters comprising the composite major adverse events end point (i.e. late coronary revascularization, cardiac hospitalization, non-fatal myocardial infarction and cardiac death) between patients with and without perfusion defects. Late coronary revascularization, cardiac hospitalization and cardiac death were significantly higher in subjects with stress induced perfusion defects (log rank p-value = 0.001, = 0.004 and = 0.003 respectively). There was no significant difference between the two groups in terms of non-fatal myocardial infarction

Table 3 shows the incidence of MACE composites in patients with and without stress induced perfusion defects. Results of Kaplan Meier analysis showed that the primary endpoint of composite MACE was significantly different between patients with and without stress induced perfusion defects (p < 0.001) (Fig. 3). On sub-analysis of the individual endpoints, late coronary revascularization (p = 0.001), cardiac hospitalization (p = 0.004) and cardiac death (p = 0.003) were significantly different between the two groups (see Fig. 4a–d). There was no significant difference in non-fatal MI (p = 0.646) (see Fig. 4c).

**Table 3:** Incidence of composite endpoints in patients with and without stress inducible perfusion defects; MI, myocardial infarction

Inducible Perfusion Defect	Late coronary revascularization	Cardiac hospitalization	Non-fatal MI	Cardiac death	Total
Present	6	10	0	3	19
Absent	5	17	1	1	24
Total number	11	27	1	4	43

The annualised event rate for patients with no stress induced perfusion defect was 0.4% vs 2.8% for patients with perfusion defects (Table 4).

**Table 4:** Annualized event rates for patients with and without stress inducible perfusion defects

	Inducible perfusion defect present (n = 38)	Inducible perfusion defect absent (n = 170)
Total number of non-fatal MI and cardiac death	3	2
Total patient years	108.0	544.9
Annualized event rate (per patient year)	2.8%	0.4%

*MI* myocardial infarction

### Predictors for higher incidence rate of MACE

Subjects with MACE had significantly higher age, smoking rates, estimated glomerular filtration rate, prevalence of atrial fibrillation, LV end-diastolic volume index, LV end-systolic volume index, LV mass index, resting heart rate, LVEF < 50%/ lower LVEF, infarct detected by LGE, stress induced perfusion defect (see Table 1). Univariate Cox regression analysis of these factors is shown in Additional file 1: Table S2.

Using multivariable Cox regression analysis, stress induced perfusion defect (p < 0.001, hazard ratio [HR] = 3.63), lower LVEF (p < 0.001, HR = 0.96) and infarct detected by LGE (p = 0.001 h = 2.92) were identified as the predictors for higher incidence of MACE (Table 5).

**Table 5:** Multivariable Cox regression model with stress induced perfusion defect, presence of LGE infarct and LVEF as variables. LVEF is a continuous variable

	Coefficient	p-value	Hazard ratio	95% CI for Hazard ratio	
				(Lower)	(upper)
Stress induced perfusion defect	1.289	< 0.001	3.630	1.879	7.012
LVEF	-0.037	< 0.001	0.964	0.945	0.984
LGE Infarct	1.073	0.001	2.924	1.517	5.634

*CI* Confidence Interval, *CMR* Cardiovascular magnetic resonance, *LGE* Late gadolinium enhancement, *LVEF* left ventricular ejection fraction

## Discussion

Our study showed that similar to other vasodilator stress agents, ATP stress CMR is also predictive of MACE. A stress induced perfusion defect on ATP stress CMR was associated with a higher risk of MACE (hazard ratio = 3.63) over a median follow-up of 3.3 years with an annualized event rate of 2.8%. The main drivers of this increased risk were the incidence of cardiac hospitalization, late coronary revascularization and cardiac death which were significantly higher in patients with stress induced perfusion defects on ATP stress CMR examinations. Alternatively, absence of a stress induced perfusion defect on ATP stress CMR had an annualized event rate of 0.4%. With multivariable cox regression analysis, stress induced perfusion defect, LVEF and MI detected by LGE were independent rick factors for MACE.

ATP has a short half-life of 20 s [23] and has vasodilatory effects like adenosine. Once ATP is infused intravenously, there is incremental cleavage of each phosphate compound. The resulting adenosine thus activates the A_1_ and A_2_ receptors producing the same vasodilatory effect as the more established intravenous adenosine infusion [24].

ATP is a readily accessible and less expensive in parts of the Asia Pacific region relative to other stress agents. The cost of ATP in our centre is approximately US$15 per patient whilst adenosine costs nearly US$70 per patient. An additional hurdle we have faced is the difficulty in obtaining adenosine and regadenoson in mainland China due to licensing and manufacturing issues. As such, centres in the Asia–Pacific frequently use ATP but the data supporting its use in CMR is not as extensive as other stress agents such as dobutamine, adenosine, dipyridamole and regadenoson [8, 18, 25]. Another issue with ATP is that centres in the Asia–Pacific region have sometimes mistaken adenosine and ATP as being the same agent. The current Society for Cardiovascular Magnetic Resonance guidelines has recently included ATP as a stress agent [26, 27] but the data supporting the imaging protocol are very limited. Evidence and drug availability are crucial in the development of stress CMR services as some countries in this region have limited access to well established stress agents (i.e. adenosine, dipyridamole and regadenoson) due to licensing issues, cost and production. Thus, this study provides timely evidence for the utilization of ATP as a stress agent for stress CMR. So far, data supporting the use of ATP has been primarily in nuclear myocardial perfusion imaging studies and the protocols have been adapted for stress CMR [11, 14].

This study also demonstrates a safe and clinically feasible ATP protocol with a starting infusion rate of 0.14 mg/kg/min in which no patients experienced significant complications. The most common side effects of ATP infusion in our study were shortness of breath, chest pain and headache which largely agree with a previous report [13]. However, these side effects were mild and resolved within 5 min after ATP infusion was stopped. Indeed, we also demonstrated the feasibility of increasing the ATP infusion rate by 50% in patients not responding adequately. This 50% increase in infusion rate has been safely demonstrated in adenosine stress CMR previously [17] but not in ATP stress CMR. Some studies have suggested that ATP should be given at slightly higher infusion rates of 0.16 mg/kg/min initially [24]. However, our study shows that with an infusion rate of 0.14 mg/kg/min 94.2% of patients are adequately stressed.

Compared to other studies assessing the prognostic significance of stress CMR, our study showed similar findings of increased MACE in patients with stress induced perfusion defects on stress CMR [8, 18]. Furthermore, we showed that a normal ATP stress CMR indicates a lower likelihood of MACE and adds to the growing literature that stress CMR has significant prognostic value with different pharmacological stress agents [2, 10, 28]. Thus, the choice of pharmacological stress agent should be dependent on a center’s previous experience, the availability of the pharmacological agent and the cost implications for health care.

In our study, stress induced perfusion defect, lower LVEF and LGE detected infarcts were independent predictors of MACE. Our finding of LGE and stress induced perfusion defect as independent predictors of MACE is consistent with previous publications by Freed et al. and Pontone et al. which looked at regadenoson and dipyridamole respectively [8, 18].

### Limitations

Our study has limitations. Firstly, this is a retrospective study in a Chinese population. Further research is needed to determine if this is generalizable to other populations worldwide. Secondly, our follow-up period is relatively short with relatively small number of patients with stress perfusion defects and a smaller number of patients with hard cardiovascular events. Thus, non-fatal MI although not significant in this study may actually be significant if the study length was increased and the number of subjects also increased. Nonetheless, our study still showed the prognostic value of ATP stress CMR for adverse cardiovascular events in Chinese population and data supporting the use of ATP stress CMR is required in this region to support the practice and development of stress CMR. Thirdly, we do not have other vasodilator agents like adenosine or dipyridamole for comparison to see if ATP is a comparable stress agent to more well-established stress agents for CMR. Lastly, not all patients underwent catheter/invasive coronary angiography to confirm the presence of obstructive CAD. Thus, a stress induced perfusion defect likely led to the patients undergoing catheter coronary angiography, however, the decision to revascularize was decided during the interventional procedure. In addition, our study follows previous studies in establishing the prognosis by not catheterizing all patients undergoing stress CMR [5, 8, 18].

## Conclusion

ATP stress CMR has significant prognostic value. An abnormal ATP stress CMR with findings of stress-induced perfusion defect is predictive of higher MACE events. Patients with suspected obstructive CAD without a stress induced perfusion defect on ATP stress CMR have an annualized event rate of 0.4% versus 2.8% if a stress induced perfusion defect is present.

## Supplementary Information

**Additional file 1: Table S1.** Patient characteristics of study population (without stress perfusion defect vs with stress perfusion defect). **Table S2.** Univariate Cox regression.

## Data Availability

The datasets used and/or analysed during the current study are available from the corresponding author on reasonable request.

## Abbreviations

ATP: Adenosine triphosphate
bSSFP: Balanced steady-state free precession
CAD: Coronary artery disease
CMR: Cardiovascular magnetic resonance
HR: Hazard ratio
LAD: Left anterior descending coronary artery
LCX: Left circumflex coronary artery
LGE: Late gadolinium enhancement
LV: Left ventricle/left ventricular
LVEF: Left ventricular ejection fraction
MACE: Major adverse cardiovascular events
MI: Myocardial infarction
PSIR: Phase sensitive inversion recovery
RCA: Right coronary artery
TE: Echo time
TR: Repetition time

## References

[1] Greenwood JP, Maredia N, Younger JF, Brown JM, Nixon J, Everett CC (2012). Cardiovascular magnetic resonance and single-photon emission computed tomography for diagnosis of coronary heart disease (CE-MARC): a prospective trial. Lancet.

[2] Nagel E, Greenwood JP, McCann GP, Bettencourt N, Shah AM, Hussain ST (2019). Magnetic resonance perfusion or fractional flow reserve in coronary disease. N Engl J Med.

[3] Shah R, Heydari B, Coelho-Filho O, Murthy VL, Abbasi S, Feng JH (2013). Stress cardiac magnetic resonance imaging provides effective cardiac risk reclassification in patients with known or suspected stable coronary artery disease. Circulation.

[4] Jahnke C, Nagel E, Gebker R, Kokocinski T, Kelle S, Manka R (2007). Prognostic value of cardiac magnetic resonance stress tests: adenosine stress perfusion and dobutamine stress wall motion imaging. Circulation.

[5] Steel K, Broderick R, Gandla V, Larose E, Resnic F, Jerosch-Herold M (2009). Complementary prognostic values of stress myocardial perfusion and late gadolinium enhancement imaging by cardiac magnetic resonance in patients with known or suspected coronary artery disease. Circulation.

[6] Greenwood JP, Herzog BA, Brown JM, Everett CC, Nixon J, Bijsterveld P, et al. Prognostic value of cardiovascular magnetic resonance and single-photon emission computed tomography in suspected coronary heart disease: Long-term follow-up of a prospective, diagnostic accuracy cohort study. Ann Intern Med. 2016;165:1–9. 10.7326/M15-1801 27158921

[7] Kazmirczak F, Nijjar PS, Zhang L, Hughes A, Chen K-HA, Okasha O (2019). Safety and prognostic value of regadenoson stress cardiovascular magnetic resonance imaging in heart transplant recipients. J Cardiovasc Magn Reson..

[8] Pontone G, Andreini D, Bertella E, Loguercio M, Guglielmo M, Baggiano A (2016). Prognostic value of dipyridamole stress cardiac magnetic resonance in patients with known or suspected coronary artery disease: a mid-term follow-up study. Eur Radiol.

[9] Gaibazzi N, Reverberi C, Lorenzoni V, Molinaro S, Porter TR (2012). Prognostic value of high-dose dipyridamole stress myocardial contrast perfusion echocardiography. Circulation.

[10] Lipinski MJ, McVey CM, Berger JS, Kramer CM, Salerno M (2013). Prognostic value of stress cardiac magnetic resonance imaging in patients with known or suspected coronary artery disease: a systematic review and meta-analysis. J Am Coll Cardiol.

[11] Chun KA, Lee J, Lee SW, Ahn BC, Ha JH, Cho IH (2006). Direct comparison of adenosine and adenosine 5'-triphosphate as pharmacologic stress agents in conjunction with Tl-201 SPECT: Hemodynamic response, myocardial tracer uptake, and size of perfusion defects in the same subjects. J Nucl Cardiol.

[12] Watanabe K, Sekiya M, Ikeda S, Miyagawa M, Kinoshita M, Kumano S (1997). Comparison of adenosine triphosphate and dipyridamole in diagnosis by thallium-201 myocardial scintigraphy. J Nucl Med.

[13] Garcia-Baizan A, Millor M, Bartolome P, Ezponda A, Pueyo JC, Gavira JJ (2019). Adenosine triphosphate (ATP) and adenosine cause similar vasodilator effect in patients undergoing stress perfusion cardiac magnetic resonance imaging. Int J Cardiovasc Imaging.

[14] Yao Z, Zhu H, Li W, Chen C, Wang H, Shi L (2017). Adenosine triphosphate stress myocardial perfusion imaging for risk stratification of patients aged 70 years and older with suspected coronary artery disease. J Nucl Cardiol.

[15] Bravo N, Gimenez M, Mejia S, Garcia-Velloso MJ, Coma-Canella I (2002). Prognostic value of myocardial perfusion imaging with adenosine triphosphate. J Nucl Cardiol.

[16] Coma-Canella I, Palazuelos J, Bravo N, Garcia Velloso MJ (2006). Myocardial perfusion imaging with adenosine triphosphate predicts the rate of cardiovascular events. J Nucl Cardiol.

[17] Karamitsos TD, Ntusi NA, Francis JM, Holloway CJ, Myerson SG, Neubauer S (2010). Feasibility and safety of high-dose adenosine perfusion cardiovascular magnetic resonance. J Cardiovasc Magn Reson.

[18] Freed BH, Narang A, Bhave NM, Czobor P, Mor-Avi V, Zaran ER (2013). Prognostic value of normal regadenoson stress perfusion cardiovascular magnetic resonance. J Cardiovasc Magn Reson.

[19] Vincenti G, Masci PG, Monney P, Rutz T, Hugelshofer S, Gaxherri M (2017). Stress Perfusion CMR in patients with known and suspected CAD: prognostic value and optimal ischemic threshold for revascularization. JACC Cardiovasc Imaging.

[20] Ng M-Y, Zhou W, Vardhanabhuti V, Lee C-H, Yu EYT, Wan EYF (2020). Cardiac magnetic resonance for asymptomatic patients with type 2 diabetes and cardiovascular high risk (CATCH): a pilot study. Cardiovasc Diabetol.

[21] Tong X, Li V, Liu A, So E, Chan Q, Ho K, et al. Cardiac Magnetic Resonance T1 Mapping in Adolescent and Young Adult Survivors of Childhood Cancers: A Case-Control Study. Circ Cardiovasc Imag. 2019. 10.1161/CIRCIMAGING.118.008453 30929466

[22] Mosteller RD (1987). Simplified calculation of body-surface area. N Engl J Med.

[23] Jeremias A, Filardo SD, Whitbourn RJ, Kernoff RS, Yeung AC, Fitzgerald PJ (2000). Effects of intravenous and intracoronary adenosine 5'-triphosphate as compared with adenosine on coronary flow and pressure dynamics. Circulation.

[24] Miyagawa M, Kumano S, Sekiya M, Watanabe K, Akutzu H, Imachi T (1995). Thallium-201 myocardial tomography with intravenous infusion of adenosine triphosphate in diagnosis of coronary artery disease. J Am Coll Cardiol.

[25] Jahnke C, Furundzija V, Gebker R, Manka R, Frick M, Schnackenburg B (2012). Gender-based prognostic value of pharmacological cardiac magnetic resonance stress testing: head-to-head comparison of adenosine perfusion and dobutamine wall motion imaging. Int J Cardiovasc Imaging.

[26] Kramer CM, Barkhausen J, Bucciarelli-Ducci C, Flamm SD, Kim RJ, Nagel E (2020). Standardized cardiovascular magnetic resonance imaging (CMR) protocols: 2020 update. J Cardiovasc Magn Reson.

[27] Kramer CM, Barkhausen J, Flamm SD, Kim RJ, Nagel E, Society for Cardiovascular Magnetic Resonance Board of Trustees Task Force on Standardized P. Standardized cardiovascular magnetic resonance (CMR) protocols 2013 update. J Cardiovasc Magn Reson. 2013;15(1):91.

[28] Gargiulo P, Dellegrottaglie S, Bruzzese D, Savarese G, Scala O, Ruggiero D (2013). The prognostic value of normal stress cardiac magnetic resonance in patients with known or suspected coronary artery disease: a meta-analysis. Circ Cardiovasc Imaging.

