# Supplementary material for: Prognostic value of perfusion cardiovascular magnetic resonance with adenosine triphosphate stress in stable coronary artery disease
Source: J Cardiovasc Magn Reson. 2021 Jun 24;23:75. doi: 10.1186/s12968-021-00770-z (PMC8223349; doi:10.1186/s12968-021-00770-z)
Supplement: Supplementary file 1 — Additional file 1: Table S1. Patient characteristics of study population (without stress perfusion defect vs with stress perfusion defect). Table S2. Univariate Cox regression. [file 12968_2021_770_MOESM1_ESM.docx]

**Supplementary Tables**

|  | **Subjects without stress perfusion defect**  **(n=170)** | **Subjects with stress perfusion defect**  **(n=38)** | **P value** |
| --- | --- | --- | --- |
| **General information** | | | |
| Age (yrs) | 60.6±15.0 | 64.0±13.6 | 0.201 |
| Male | 98 (57.6) | 25 (65.8) | 0.356 |
| Height (cm) | 163.6±9.7 | 163.3±10.3 | 0.895 |
| Weight (kg) | 67.3±14.1 | 67.3±15.5 | 0.995 |
| BMI (m^2^) | 25.0±4.2 | 25.0±4.0 | 0.946 |
| Hypertension | 89 (52.4) | 27 (71.1) | 0.036* |
| Diabetes | 43 (25.3) | 10 (26.3) | 0.896 |
| Hyperlipidemia | 60 (35.3) | 18 (47.4) | 0.165 |
| Smoking | 16 (9.4) | 4 (10.5) | 0.833 |
| Glomerular filtration rate (mL/min/1.73m^2^) | 80.1±21.6 | 74.8±23.0 | 0.216 |
| Atrial fibrillation | 9 (5.3) | 5 (13.2) | 0.082 |
| Ventricular Ectopic | 13 (7.6) | 1 (2.6) | 0.262 |
| **Cardiac history** | | | |
| Congestive heart failure | 10 (5.9) | 1 (2.6) | 0.418 |
| Myocardial infarction | 0 | 2 (5.3) | 0.003* |
| Coronary artery disease | 53 (31.2) | 18 (47.4) | 0.057 |
| **Symptoms for CMR referral** | | | |
| Chest pain | 70 (41.2) | 14 (36.8) | 0.623 |
| Shortness of breath | 8 (4.7) | 0 | 0.173 |
| Palpitation | 6 (3.5) | 0 | 0.240 |
| Dizziness | 2 (1.2) | 0 | 0.502 |
| Loss of consciousness | 3 (1.8) | 1 (2.6) | 0.725 |
| **CMR parameters** | | | |
| 1.5T | 76 (44.7) | 11 (28.9) | 0.075 |
| 3.0T | 94 (55.3) | 27 (71.2) | 0.075 |
| LV end-diastolic volume index(mL/m^2^) | 84.1±30.2 | 87.8±50.1 | 0.549 |
| LV end-systolic volume index(mL/m^2^) | 37.1±29.6 | 44.2±51.1 | 0.251 |
| LV ejection fraction (%) | 59.6±14.5 | 55.7±14.8 | 0.136 |
| LV mass index (g/m^2^) | 63.7±22.9 | 68.1±26.4 | 0.397 |
| HR at rest (bpm) | 68.4±13.8 | 70.4±12.7 | 0.431 |
| SBP at rest (mmHg) | 141.1±20.5 | 140.6±23.7 | 0.900 |
| DBP at rest (mmHg) | 85.1±12.9 | 91.5±12.3 | 0.127 |
| ATP infusion time (min) | 4.4±0.8 | 4.2±0.7 | 0.069 |
| Abnormal wall motion | 25 (14.7) | 14 (36.8) | 0.002* |
| **Medications** | | | |
| Beta-blocker | 73 (42.9) | 15 (39.5) | 0.696 |
| Ca-channel blocker | 50 (29.4) | 11 (28.9) | 0.955 |
| ACE inhibitor | 41 (24.1) | 11 (28.9) | 0.534 |
| Statin | 97 (57.1) | 26 (68.4) | 0.198 |
| Aspirin | 82 (48.2) | 20 (52.6) | 0.624 |
| Digoxin | 1 (0.6) | 0 | 0.636 |
| **Side-effects** | | | |
| Chest pain | 50 (29.4) | 14 (36.8) | 0.370 |
| Shortness of breath | 29 (17.1) | 12 (31.6) | 0.042* |
| Headache | 12 (7.1) | 5 (13.2) | 0.215 |
| Palpitation | 13 (7.6) | 2 (5.3) | 0.608 |
| Hot flushing | 2 (1.2) | 0 | 0.502 |
| **Stress CMR findings** | | | |
| LVEF <50% | 25 (14.7) | 10 (26.3) | 0.084 |
| Myocardial LGE | 36 (21.2) | 15 (39.5) | 0.018* |
| LGE (%) | 1.13±6.35 | 2.92±7.42 | *0.009** |
| **Table S1** - Patient characteristics of study population (without stress perfusion defect vs with stress perfusion defect)  Data is presented as mean ± standard deviation or count with percentage in brackets. ATP: adenosine triphosphate; ACE: angiotensin converting enzyme; BSA: body surface area; BMI: body mass index; PCI: percutaneous coronary intervention; HR: heart rate; SBP: systolic blood pressure; DBP: diastolic blood pressure; LV = Left ventricle; LGE = Late gadolinium enhancement; CABG = Coronary artery bypass graft  * = p<0.05 | | | |

|  | HR | 95% CI | P value |
| --- | --- | --- | --- |

| Age | 1.047 | 1.019-1.076 | 0.001 |
| --- | --- | --- | --- |
| Smoking | 3.072 | 1.458-6.474 | 0.003 |
| GFR (mL/min/1.73 m^2^) | 0.982 | 0.967-0.998 | 0.029 |
| Atrial fibrillation | 4.495 | 2.062-9.799 | *<0.001* |
| LV end-diastolic volume index(mL/m^2^) | 1.015 | 1.008-1.023 | *<0.001* |
| LV end-systolic volume index(mL/m^2^) | 1.017 | 1.011-1.023 | *<0.001* |
| LV ejection fraction (%) | 0.960 | 0.944-0.978 | *<0.001* |
| LV mass index (g/m^2^) | 1.029 | 1.016-1.042 | *<0.001* |
| HR at rest (bpm) | 1.044 | 1.023-1.065 | *<0.001* |
| Abnormal wall motion | 5.068 | 2.598-9.762 | *<0.001* |
| LGE infarct | 4.339 | 2.306-8.164 | *<0.001* |
| Stress Induced Perfusion Defect | 4.525 | 2.379-8.608 | *<0.001* |
| **Table S2.** Univariate Cox regression  OR = Hazard ratio  CI = Confidence Interval | | | |
